# Identification of raw as a regulator of glial development

**DOI:** 10.1371/journal.pone.0198161

**Published:** 2018-05-29

**Authors:** Diana Luong, Luselena Perez, Jennifer C. Jemc

**Affiliations:** Loyola University Chicago, Department of Biology, Chicago, Illinois, United States of America; University of Dayton, UNITED STATES

## Abstract

Glial cells perform numerous functions to support neuron development and function, including axon wrapping, formation of the blood brain barrier, and enhancement of synaptic transmission. We have identified a novel gene, *raw*, which functions in glia of the central and peripheral nervous systems in *Drosophila*. Reducing Raw levels in glia results in morphological defects in the brain and ventral nerve cord, as well as defects in neuron function, as revealed by decreased locomotion in crawling assays. Examination of the number of glia along peripheral nerves reveals a reduction in glial number upon *raw* knockdown. The reduced number of glia along peripheral nerves occurs as a result of decreased glial proliferation. As Raw has been shown to negatively regulate Jun N-terminal kinase (JNK) signaling in other developmental contexts, we examined the expression of a JNK reporter and the downstream JNK target, *matrix metalloproteinase 1* (*mmp1*), and found that *raw* knockdown results in increased reporter activity and Mmp1 levels. These results are consistent with previous studies showing increased Mmp levels lead to nerve cord defects similar to those observed upon *raw* knockdown. In addition, knockdown of *puckered*, a negative feedback regulator of JNK signaling, also causes a decrease in glial number. Thus, our studies have resulted in the identification of a new regulator of gliogenesis, and demonstrate that increased JNK signaling negatively impacts glial development.

## Introduction

The precise interaction between neurons and glia is essential for the establishment and maintenance of nervous system structure and function. A failure of neurons and glia to interact properly or a breakdown in interactions can result in neuropathy or neurodegeneration [1–3]. Neurons play a critical role in sensing environmental stimuli and conveying responses to these stimuli. However, their function depends on their close association with different types of glia. Glia perform a variety of roles in the nervous system, from regulating blood brain barrier (BBB) formation and synapse structure to insulating neurons, clearing debris, and providing trophic support (reviewed in [4]).

**Fig 1:**
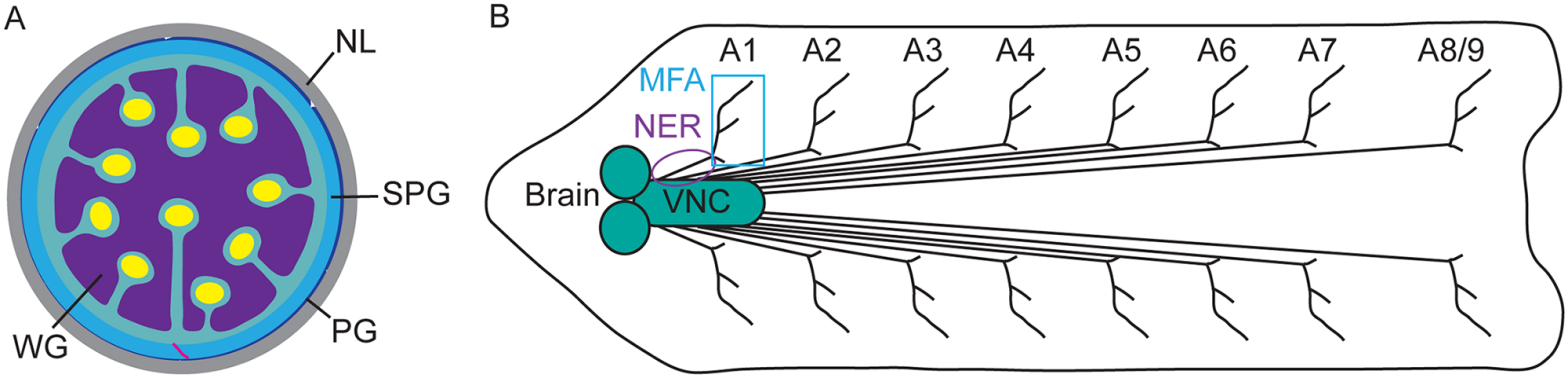
Third instar larval nervous system structure. (A) Cross section of a peripheral nerve. Axons are in yellow, wrapping glia (WG) in purple, subperineurial glia (SPG) in light blue, perineurial glia (PG) in dark blue, septate junctions in pink, and neural lamella (NL) in gray. (B) Schematic of the third instar larval nervous system. The brain, ventral nerve cord (VNC) and peripheral nerves A1-A8/9 are illustrated with the muscle field area (MFA) boxed and the nerve extension region (NER) encircled.

*Drosophila melanogaster* has proven a fruitful organism for studying the roles of different glial subtypes and the molecular mechanisms that regulate their development and function, given the availability of genetic tools to manipulate gene expression in glia and the ability to visualize glia throughout development. Glia and neurons are derived from a common population of precursor cells, known as neural stem cells, or neuroblasts in *Drosophila* [5]. During embryogenesis, neuroblasts in the ventral nerve cord (VNC) give rise to embryonic peripheral glia (ePG), which migrate out of the central nervous system (CNS) along peripheral nerves, resulting in the presence of 12 ePG in each abdominal hemisegment [5, 6]. The 12 ePG are located in approximately the same position in each hemisegment [7]. Each of these ePG then differentiates, forming the three glial layers that ensheath larval peripheral nerves (Fig 1A) [6]. Wrapping glia, which are derived from ePG1, 5, and 9, form the innermost layer and begin ensheathing axon bundles during early larval stages and continue to ensheath individual axons in the third larval stage, separating sensory neurons from motor neurons [6, 8]. Subperineurial glia, which are derived from ePG3, 4, 7, and 10, form the middle layer and are responsible for formation of the BBB in *Drosophila*, sealing off the nerves from the hemolymph via the formation of septate junctions (*Drosophila* tight junctions) [6, 8–10]. This barrier regulates ion homeostasis and controls the influx of nutrients into the CNS and the efflux of waste [11]. Perineurial glia, which are derived from ePG2, 6, and 8, form the outermost layer in the third larval stage, and are believed to provide trophic support [6, 8]. These perineurial glia are surrounded by a neural-specific extracellular matrix (ECM), known as the neural lamella, which is present from embryonic stage 16 onward and appears continuous between the peripheral nervous system (PNS) and CNS [8]. The neural lamella is critical for the wrapping of both the CNS and the peripheral nerves [12, 13].

Lineage tracing of ePG revealed that all 12 ePG survive until at least the third larval stage, with ePG1-3 localizing to the nerve extension region (NER) of peripheral nerves and ePG4-12 localizing to the muscle field area (MFA) (Fig 1B) [6]. Along the NER, ePG2 is the only mitotically active glial cell during larval stages, proliferating to give rise to all larval perineurial glia in this region in a manner that is proportional to the surface area of the nerve [6, 14]. Subperineurial glia undergo endoreplication, resulting in polyploidy, which is critical for the increase in cell size needed for the establishment of the BBB [15, 16].

Previous studies have provided significant insight into the molecules expressed in different glial subtypes within the PNS [17]; however, understanding of the signaling pathways regulating expression of these genes and how these pathways mediate glia-glia, glia-neuron, and glia-ECM interactions to promote glial development remains far from complete. In these studies we identify the protein Raw as a critical regulator of glial development. Previous work demonstrated that Raw is required for organization of the PNS and for CNS retraction, dorsal closure, and morphogenesis of the salivary glands, hindgut, and malpighian tubules during embryogenesis, as well as larval dendritic patterning [18–20]. In addition, Raw is necessary for the ensheathment of germ cells (GCs) by somatic gonadal precursor cells (SGPs) in the developing gonad, a process required for proper GC proliferation and gene expression [21–23]. Structurally, Raw is a transmembrane protein with an N-terminal extracellular domain and a C-terminal intracellular domain that lacks additional domains [19]. Analysis of the molecular context of Raw function during embryogenesis revealed that Raw negatively regulates Jun N-terminal Kinase (JNK) signaling and positively regulates cell adhesion through *Drosophila* E-Cadherin [20, 22, 24]. More recent studies have shown that Raw also interacts with the Nuclear dbf2-related (NDR) family kinase, Tricornered (Trc), in dendrites [19].

The requirement of Raw for the ensheathment of GCs by SGPs in the developing gonad and the previously described role of Raw in embryonic nervous system development led us to ask if Raw may play a specific role in glia to promote nervous system development. In addition, we were curious if Raw may function as a negative regulator of JNK signaling in the glia as it does in other contexts. This study demonstrates that Raw is required for proper glial development and the establishment of nervous system structure and function. Reducing *raw* levels in glia results in abnormal brain and VNC structure, as well as a reduced number of glia along peripheral nerves. Further exploration of these phenotypes reveals that Raw is required for glial proliferation, and negatively regulates JNK signaling to promote proper glial development.

## Materials and methods

### Fly strains and genetics

The following fly stocks were obtained from the Bloomington *Drosophila* Stock Center (Indiana University, Bloomington, IN, U.S.A): UAS-*raw*-*RNAi*^3^ (BL#31393; [25]), *repo*-Gal4 (BL#7415), and UAS-*puc-RNAi* (BL#57300; [26]). *w*^*1118*^ (Mark Van Doren), *repo*-Gal4, UAS-monomeric Red Fluorescent Protein (mRFP; Catherine Collins), UAS-*raw-RA* (cDNA as described in [22] inserted into the *attp2* site on the 3rd chromosome; referred to as UAS-*raw* throughout this paper), TRE-GFP (tetradecanoylphorbol acetate response element-Green Fluorescent Protein; a JNK reporter containing four Activator Protein-1 (AP-1) binding sites (Dirk Bohmann [27])), UAS-*dicer2* (*dcr2*) [28], and UAS-*raw*-*RNAi*^*2*^ (#24532; Vienna *Drosophila* Resource Center; [28]) stocks were also utilized. Mutations were maintained over TM6B, Yellow Fluorescent Protein- or GFP-marked balancer chromosomes in order to select for larvae of the correct genotype.

In order to alter levels of *raw* and *puc* specifically in the glia, the Gal4/UAS system was utilized to promote knockdown via RNAi. The following genotypes were analyzed: 1) *repo*-Gal4, UAS-*mRFP*; 2) UAS-*raw*-*RNAi*^*2*^; *repo*-Gal4, UAS-*mRFP*; 3) *repo*-Gal4, UAS-*mRFP*/UAS-*raw*-*RNAi*^*3*^; 4) *repo*-Gal4, UAS-*mRFP*/UAS-*dicer2*; 5) UAS-*raw*-*RNAi*^*2*^; *repo*-Gal4, UAS-*mRFP*/UAS-*dicer2*; 6) *repo*-Gal4, UAS-*mRFP*/UAS-*raw*; 7) UAS-*raw*-*RNAi*^*2*^; *repo*-Gal4/UAS-*raw*; and 8) *repo*-Gal4, UAS-*mRFP*/UAS-*puc-RNAi*. Note that UAS-*mRFP* was present in all genotypes analyzed with the exception of #7 above. All experiments were performed at 25°C.

### Immunohistochemistry and image analysis

For analysis of the PNS, wandering third instar larvae were dissected in 1x phosphate-buffered saline (PBS) and fixed in 4% paraformaldehyde (PFA) for 20 minutes. Following washing in PBS + 0.1% Triton X-100 (PBTx), samples were blocked for 1 hour in PBTx + 5% normal goat serum (NGS). For eye imaginal discs, wandering third instar larvae were dissected in 1x PBS and fixed in 4% PFA +0.1% Triton for 10 minutes. Samples were washed in PBTx and blocked for 1 hour in PBTx + 1% NGS. The following primary antibodies were used: mouse anti-Reversed polarity (Repo; 1:10; Developmental Studies Hybridoma Bank (DSHB)), rabbit anti-Horseradish Peroxidase (HRP; 1:200; Jackson ImmunoResearch), rabbit anti-phospho-Histone H3 (pH3; 1:100–1:200; Cell Signaling Technology [29]), rabbit anti-cleaved *Drosophila* Death caspase-1 (Dcp-1; 1:10; Cell Signaling Technology [30]), chick anti-GFP (1:1000; abcam); rabbit anti-Red Fluorescent Protein (RFP: 1:1000; Rockland), rat anti-Elav (1:50, DSHB), and mouse anti-Matrix metalloproteinase 1 (Mmp1; 1:1:1 mix of 3A6B4, 5H7B11, and 3B8D12; mix used at 1:10; DSHB [31]). Secondaries were goat anti-rabbit, goat anti-rat, goat anti-chick or goat anti-mouse conjugated to Alexa 488, Alexa 555, or Alexa 633 (Invitrogen) used at 1:500. Samples were mounted in DABCO (70% glycerol, 2.5% 1,4-diazabicyclo[2.2.2]octane, 10mM Tris-HCl pH 7.5) and imaged using an Olympus Fluoview 1000 confocal microscope equipped with 488, 561, and 633 lasers. Images were processed using ImageJ. The number of glial nuclei along the NER of the A8/9 peripheral nerves was counted. For analysis of glial proliferation, the % of mitotic glia was calculated as the % of Repo-positive nuclei that co-labeled with pH3 along the NER of the A8/9 peripheral nerves. Statistical significance of glial number and mitotic glia data was assessed using GraphPad Prism 7 by one-way ANOVA with post-hoc Tukey test.

In order to quantitate relative changes in Mmp1 and TRE-GFP reporter levels, average pixel intensity was measured over a region of the VNC and over neighboring muscle tissue, which is not expected to undergo any changes in Mmp1 or GFP reporter levels when *raw* levels are altered in glia. Average pixel intensity in the VNC was normalized to average pixel intensity in the muscle. These values were graphed and the change relative to controls was determined. In the case of Mmp1 staining, data was analyzed using GraphPad Prism 7 by one-way ANOVA with post-hoc Tukey test. In the case of the TRE-GFP reporter, data from the control versus the *raw* knockdown sample was analyzed using GraphPad Prism 7 by the Mann-Whitney test.

### Morphological analysis

For morphological analysis of the VNC, wandering third instar larvae were imaged using a Zeiss SteREO Discovery.V12 or a Zeiss Axio Zoom.V16 microscope, and their length was measured using Zeiss Zen software. The brain and VNC were dissected in 1x PBS and imaged on a Zeiss AxioImager.M2 or Olympus DSU spinning disc microscope, and the length of the VNC was measured using Zeiss Zen or Olympus Metamorph software, respectively. The length of the VNC relative to body length was calculated for each sample, as previously described, given that VNC length has been demonstrated to scale with overall body length in other insects [32, 33]. All data was analyzed using GraphPad Prism 7 by one-way ANOVA with post-hoc Tukey test to assess statistical significance.

### Locomotor analysis

Locomotor function of third instar larvae was assessed by a crawling assay. Third instar larvae were placed on a slab of 2% agarose brought to a temperature of 25°C. The agarose was placed on a piece of plexiglass and dropped from a distance of ~1–2 inches to stimulate larval movement. Larvae were imaged for 50–60 seconds with an Apple iPhone Camera. Videos were converted to avi format by ffmpeg. The resulting avi videos were analyzed from the 15–45 second mark using ImageJ software with the wrmtrck plugin [34]. Specifications were adjusted for tracking of *Drosophila* larvae. Data on path length and average speed was analyzed by Mann-Whitney test using GraphPad Prizm 7 Software.

## Results

### *raw* knockdown results in reduced glial cell number

Strong hypomorphic alleles of *raw* lead to defects in embryonic nervous system development and are embryonic lethal [18, 21]. Therefore, to further examine Raw function in the developing nervous system beyond embryogenesis, the GAL4/UAS system was utilized to knockdown *raw* levels via RNA interference (RNAi). Previous studies have demonstrated that *reversed polarity* (*repo*) is expressed in all glia with the exception of the midline glia [35]. Therefore, *repo*-Gal4 was crossed to two UAS-*raw-RNAi* lines with overlapping *raw* target regions, one inserted on the second chromosome and one inserted on the third chromosome, referred to as UAS-*raw*-*RNAi*^*2*^ and UAS-*raw*-*RNAi*^*3*^, respectively [36, 37]. In addition, *raw* levels were reduced specifically in neurons, using *elav*-Gal4. *raw* knockdown in neither neurons nor glia affected viability. However, the co-expression of UAS-*dcr2* in the presence of UAS-*raw*-*RNAi*^*2*^ in glia led to pupal lethality, suggesting a critical requirement for Raw in these cells.

**Fig 2:**
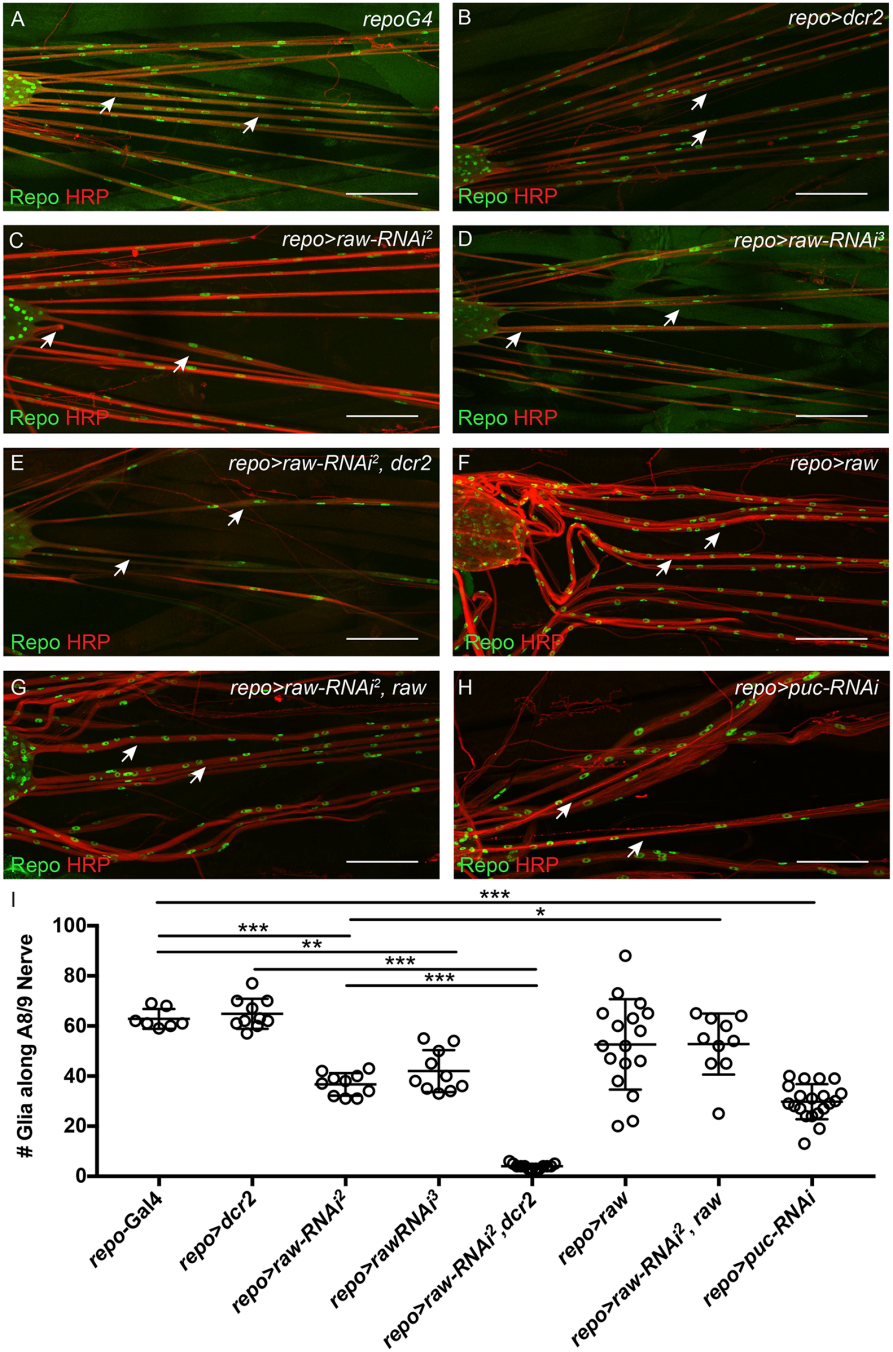
*raw* and *puc* knockdown affect glial cell number. (A-H) Maximum intensity projections of the peripheral nerves of third instar larvae. Merge of glial staining (anti-Repo; green) and neuron staining (anti-Horseradish Peroxidase; red). Arrows indicate the A8/9 nerves. Posterior to the right. (A) Control animals (*repo-Gal4*; n = 7). (B) Control animals overexpressing *dcr2* (*repo-Gal4*>*dcr2*; n = 10). (C) Panglial *raw* knockdown results in reduced glial number (*repo-Gal4*>*raw-RNAi*^*2*^; n = 10) (D) Panglial knockdown of *raw* results in reduced glial number (*repo-Gal4*>*raw-RNAi*^*3*^, n = 10). (E) Panglial *raw* knockdown in the presence of overexpressed *dcr2* results in a further reduction in glial number (*repo-Gal4*>*raw-RNAi*^*2*^, *dcr2*; n = 13). (F) Panglial *raw* overexpression does not significantly alter glial number (*repo-Gal4*>*raw*; n = 17). (G) Panglial *raw* knockdown in the presence of overexpressed *raw* rescues the *raw-RNAi* phenotype (*repo-Gal4*>*raw-RNAi*^*2*^, *raw*; n = 10). (H) Panglial *puc* knockdown results in reduced glial number (*repo-Gal4*>*puc-RNAi*; n = 19). Scale bars are 100μm. (I) Quantitation of glial number along the A8/9 peripheral nerve upon *raw* and *puc* knockdown. ***p<0.0001, **p<0.001, and *p<0.01 based on one-way ANOVA with Tukey's post hoc test.

To explore how Raw functions in glia during nervous system development, immunohistochemistry was performed on dissected third instar larvae and glial nuclei were visualized using the anti-Repo antibody. Immunostaining revealed a striking decrease in the number of glial nuclei along the NER of peripheral nerves upon *raw* knockdown. Therefore, the number of glial nuclei along the NER of the A8/9 peripheral nerves was counted in controls and *raw* knockdown larvae (Fig 2). While control larvae (*repo*-Gal4 and *repo*-Gal4>*dcr2*) had ~60–65 nuclei/nerve, this number was reduced to ~35–40 glia/nerve upon *raw* knockdown (*repo*-Gal4>*raw*-*RNAi*) using two different UAS-*raw-RNAi* lines (Fig 2). This number was further reduced to an average of less than 10 glia/nerve upon *raw* knockdown in the presence of overexpressed *dcr2* (*repo*-Gal4>*raw*-*RNAi*^*2*^, *dcr2* flies) (Fig 2). While use of the UAS-*raw-RNAi*^*3*^ line had previously been reported [36, 37], the UAS-*raw-RNAi*^*2*^ line had not been previously used in published work. Therefore, the specificity of this RNAi line was confirmed by testing the ability of overexpressed *raw* to rescue the *repo*-Gal4>*raw*-*RNAi*^*2*^ phenotype. Co-expression of *raw* significantly rescued the *raw* knockdown phenotype, increasing the average number of glial nuclei along the A8/9 nerve from 37 to 53 (Fig 2G and 2I). Overexpression of *raw* did not result in a significant change in glial number as compared to controls (*repo*-Gal4) (Fig 2F). Thus, our results demonstrate that *raw* is required for the population of nerves with the appropriate number of glial cells during development.

### Raw is required for glial proliferation and to prevent cell death along peripheral nerves

**Fig 3:**
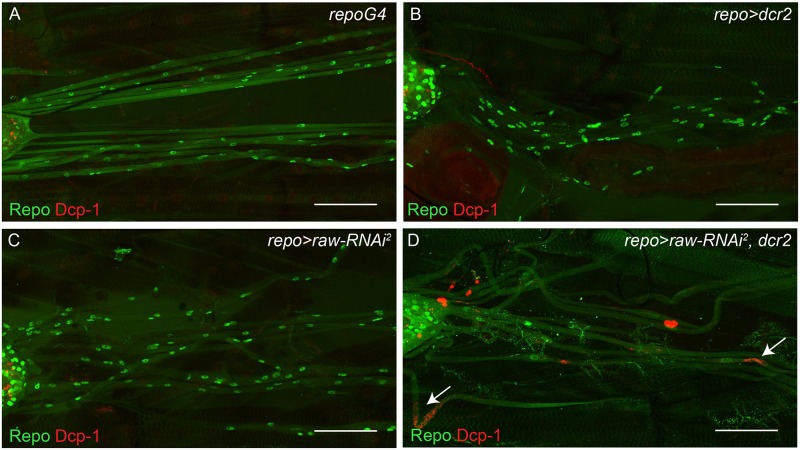
*raw* is required to prevent cell death along peripheral nerves. (A-D) Maximum intensity projections of peripheral nerves of third instar larvae co-stained for cells undergoing apoptosis (anti-cleaved Death caspase-1, red) and glia (anti-Repo, green). Posterior to the right. (A) Control animals (*repo-Gal4*; n = 8). (B) Control animals overexpressing *dcr2* (*repo-Gal4*>*dcr2*; n = 9). (C) Panglial *raw* knockdown does not increase cell death (*repo-Gal4*>*raw-RNAi*^*2*^; n = 10). (D) Panglial *raw* knockdown in the presence of overexpressed *dcr2* results in cell death along peripheral nerves (*repo-Gal4*>*raw-RNAi*^*2*^, *dcr2*; n = 9). Arrows indicate dying cells. Scale bars are 50μm.

As *raw* knockdown results in a reduced number of glia compared to controls, we hypothesized that reduced glial number could result from decreased proliferation or increased cell death along peripheral nerves in third instar larvae. Cell death was examined in *raw* knockdown and control larvae using an antibody directed against the activated form of the *Drosophila* caspase, Death caspase-1 (Dcp-1). While apoptosis of midline glia has been observed during embryogenesis and metamorphosis, apoptosis of peripheral glia has not previously been reported [38–41]. Therefore. we did not expect to observe glial cell death along nerves in controls. Consistent with this expectation, activated Dcp-1 was not observed in *repo-Gal4* controls and cell death was observed in only one out of nine *repo-Gal4*>*dcr2* samples. In contrast, *raw* knockdown in the presence of overexpressed *dcr2* (*repo*-Gal4>*raw*-*RNAi*, *dcr2* flies) resulted in the presence of Dcp-1 immunostaining along a subset of peripheral nerves in eight out of nine samples analyzed (Fig 3). This cell death only occasionally localized around Repo-positive nuclei, suggesting that reduced levels of *raw* in glia can lead to neuronal cell death. Surprisingly, cell death was not observed along nerves in *repo*-Gal4>*raw*-*RNAi* larvae (n = 10). Thus, while cell death may contribute to the decreased number of glia in third instar larvae, this does not appear to be the predominant reason for the reduction in glial number.

**Fig 4:**
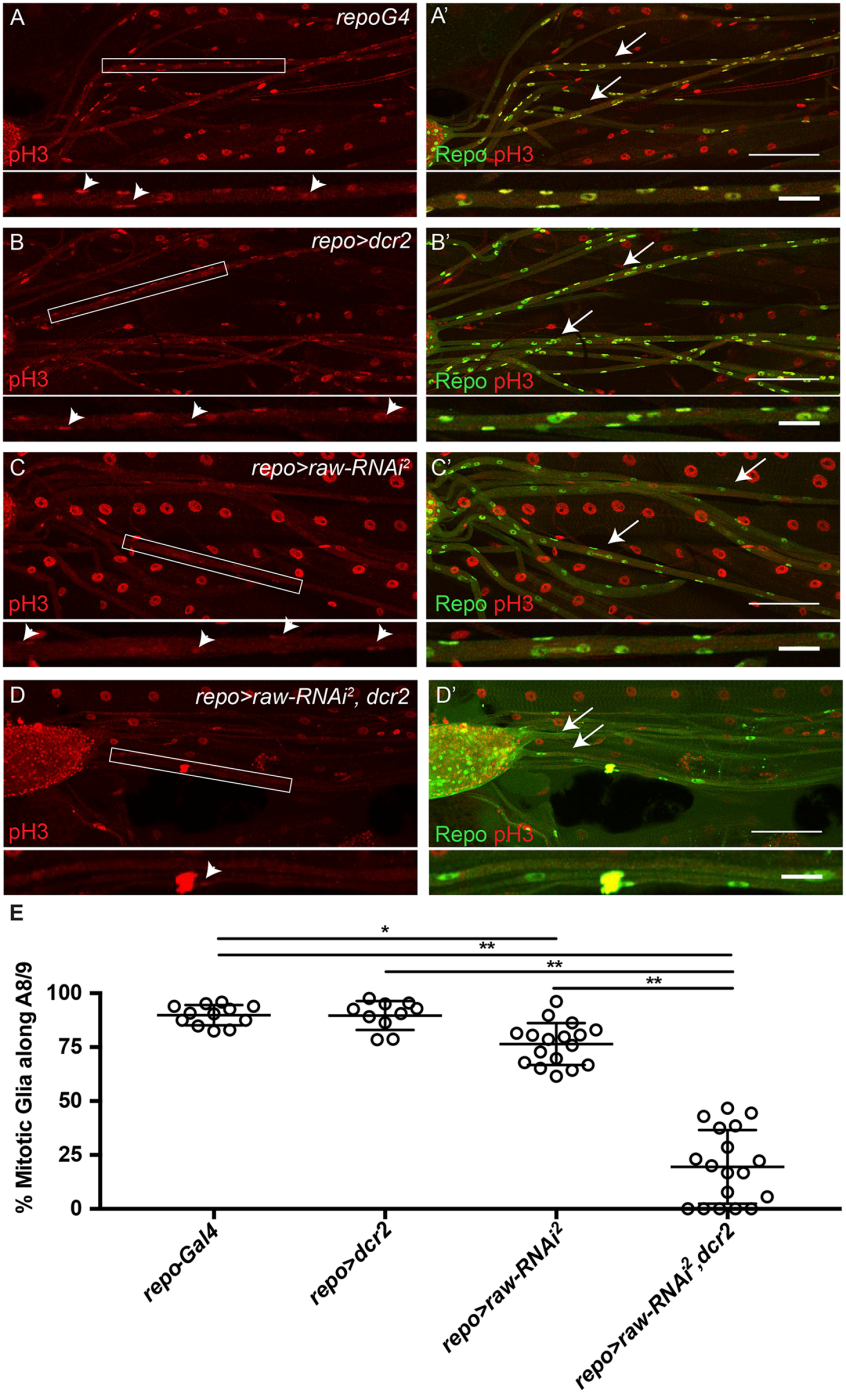
*raw* is required for glial proliferation. (A-D) Maximum intensity projections of peripheral nerves of third instar larvae stained for proliferating cells (anti-phospho-histone H3). Boxed region indicates inset shown below. Arrowheads indicate pH3-positive glial cells. (A'-D') Maximum intensity projections of peripheral nerves of third instar larvae co-stained for proliferating cells (anti-phospho-histone H3, red) and glia (anti-Repo, green). Arrows indicate A8/9 nerves. Posterior to the right. Scale bars are 100μm. For insets, scale bars are 25μm. (A, A') Control animals (*repo-Gal4*; n = 12). (B, B') Control animals overexpressing *dcr2* (*repo-Gal4*>*dcr2*; n = 10). (C, C') Panglial *raw* knockdown results in reduced glial proliferation (*repo-Gal4*>*raw-RNAi*^*2*^; n = 17). (D, D') Panglial *raw* knockdown in the presence of overexpressed *dcr2* results in a further reduction in glial proliferation (*repo-Gal4*>*raw-RNAi*^*2*^, *dcr2*; n = 18). (E) Quantitation of the % of mitotic glial cells along A8/9. *p < .05 and **p<0.0001 based on one-way ANOVA with Tukey's post hoc test.

As cell death was not observed along all nerves upon *raw* knockdown, the possibility that the decrease in glial number was due to decreased glial proliferation was examined next. Proliferation of glia along the NER of A8/9 was assessed in third instar larvae using the phospho-histone H3 (pH3) antibody, which labels mitotic cells. Specificity of the pH3 antibody was confirmed by immunostaining third instar eye imaginal discs, which revealed a pattern of pH3 immunostaining consistent with previously published studies (S1 Fig) [42]. Along the A8/9 peripheral nerve, approximately 90% of glial cells were labeled with the pH3 antibody in the *repo*-Gal4 and *repo*-Gal4>*dcr2* control samples (Fig 4). *raw* knockdown reduced the number of pH3 positive cells to just 76% of glia (*repo*-Gal4>*raw*-*RNAi*^*2*^), while *raw* knockdown in the presence of overexpressed *dcr2* (*repo*-Gal4>*raw*-*RNAi*, *dcr2* flies) further reduced the number of pH3-positive glia to ~0–50% with an average of 20% mitotic glia (Fig 4). Given that these larvae have an average of 4 glia per A8/9 nerve having one versus two mitotic glia can result in a greater range of % mitotic glia. These results suggest a reduction in the proliferation of perineurial glia, as subperineurial and wrapping glia have not been reported to proliferate along peripheral nerves.

### *raw* knockdown affects CNS morphology

**Fig 5:**
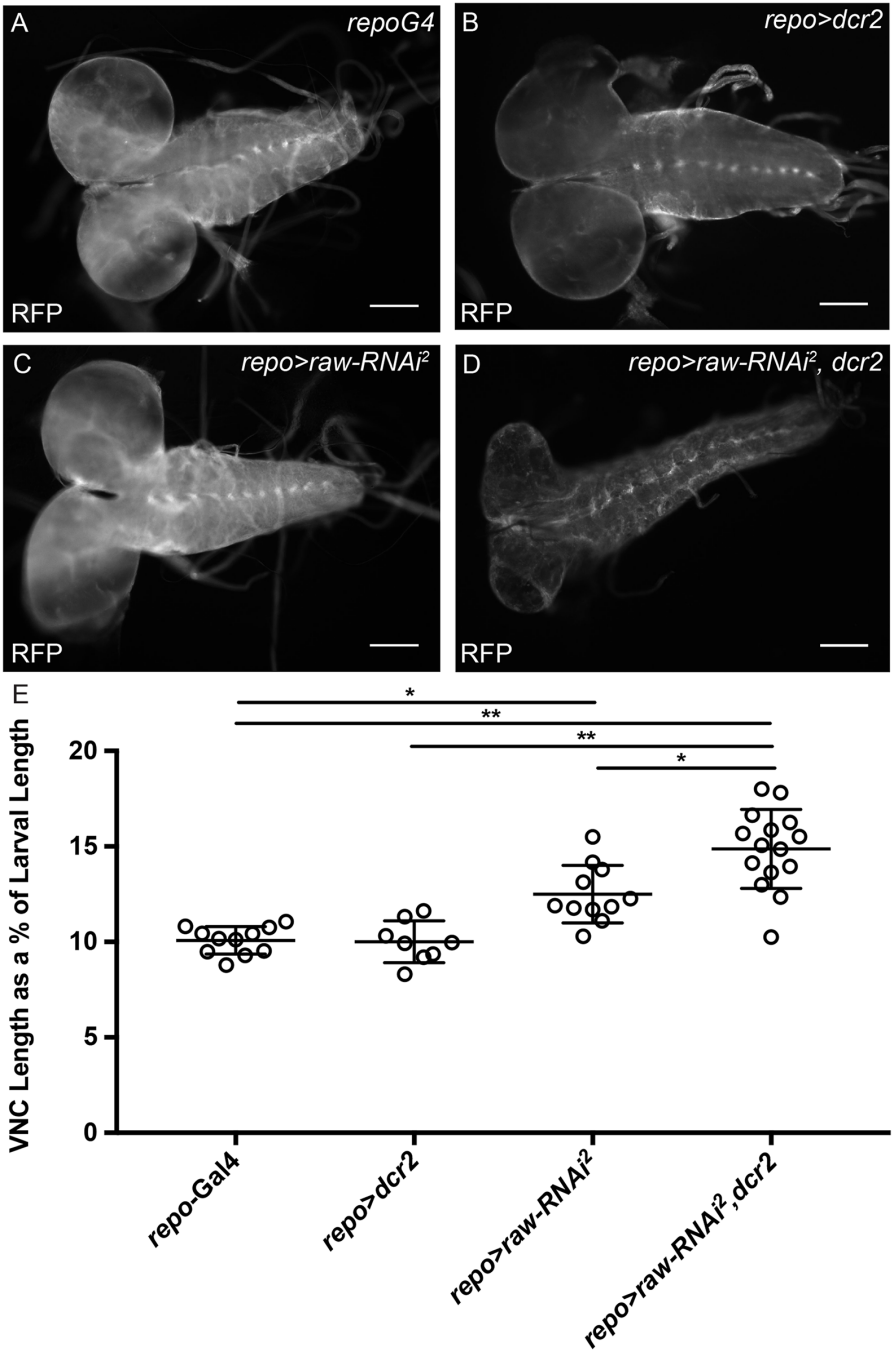
*raw* knockdown leads to an elongated nerve cord. (A-D) Images of the brain and ventral nerve cord (VNC) of third instar larvae with glia marked by expression of cytoplasmic monomeric Red Fluorescent Protein (*repo-Gal4>mRFP*). Posterior to the right. (A) Control animals (*repo-Gal4*>*mRFP* n = 11). (B) Control animals overexpressing *dcr2* do not show increased VNC length (*repo-Gal4*>*mRFP*, *dcr2*; n = 8). (C) Panglial knockdown of *raw* results in increased VNC length relative to total body length (*repo-Gal4*>UAS*-mRFP*, *raw-RNAi*^*2*^; n = 11). (D) Panglial knockdown of *raw* in the presence of overexpressed *dcr2* results in further VNC elongation relative to total body length (*repo-Gal4*>*mRFP*, *raw-RNAi*^*2*^, *dcr2*; n = 15). (E) Quantitation of ventral nerve cord length as a percentage of total body length. Scale bars are 100μm. *p < .005 and **p<0.0001 based on one-way ANOVA with Tukey's post hoc test.

In addition to defects in glial number, *raw* knockdown in the presence of overexpressed *dcr2* (*repo*-Gal4*>raw-RNAi*, *dcr2*) also caused significant defects in brain and VNC morphology. Specifically, the brain appeared smaller and the VNC appeared thin and elongated (Fig 5A–5D). Therefore, VNC length was quantified relative to total body length, as previously described, to account for age-related differences in size [33]. Quantitation revealed that nerve cords of the *repo*-Gal4 control larvae were an average of 10% of total body length (Fig 5E). Reducing *raw* levels by RNAi resulted in VNC lengths that were ~12% of total body length and this went up to ~14% of total body length when *raw* was knocked down in the presence of overexpressed *dcr2* (Fig 5E). Overexpression of *dcr2* in glia alone did not cause a significant change in the relative length of the VNC (~10% of total body length; Fig 5E). These results demonstrate a requirement for Raw in the morphological changes of the VNC during development. We also observed significant morphological differences in the brains of *repo*-Gal4*>raw-RNAi*, *dcr2* larvae compared to controls. In order to determine if brain size was reduced, the diameter of the brain was measured. A significant reduction in brain diameter was observed in *repo*-Gal4*>raw-RNAi*, *dcr2* relative to *repo*-Gal4*>dcr2* alone (d = 0.224mm versus d = 0.276mm, p<0.005), but *repo*-Gal4*>raw-RNAi* larvae did not exhibit a significant change in brain size relative to *repo>Gal4* controls (d = 0.245mm versus d = 0.245mm, not significant). These results suggest that *raw* knockdown results in significant defects in the establishment of proper nervous system morphology.

### *raw* knockdown results in increased JNK signaling

**Fig 6:**
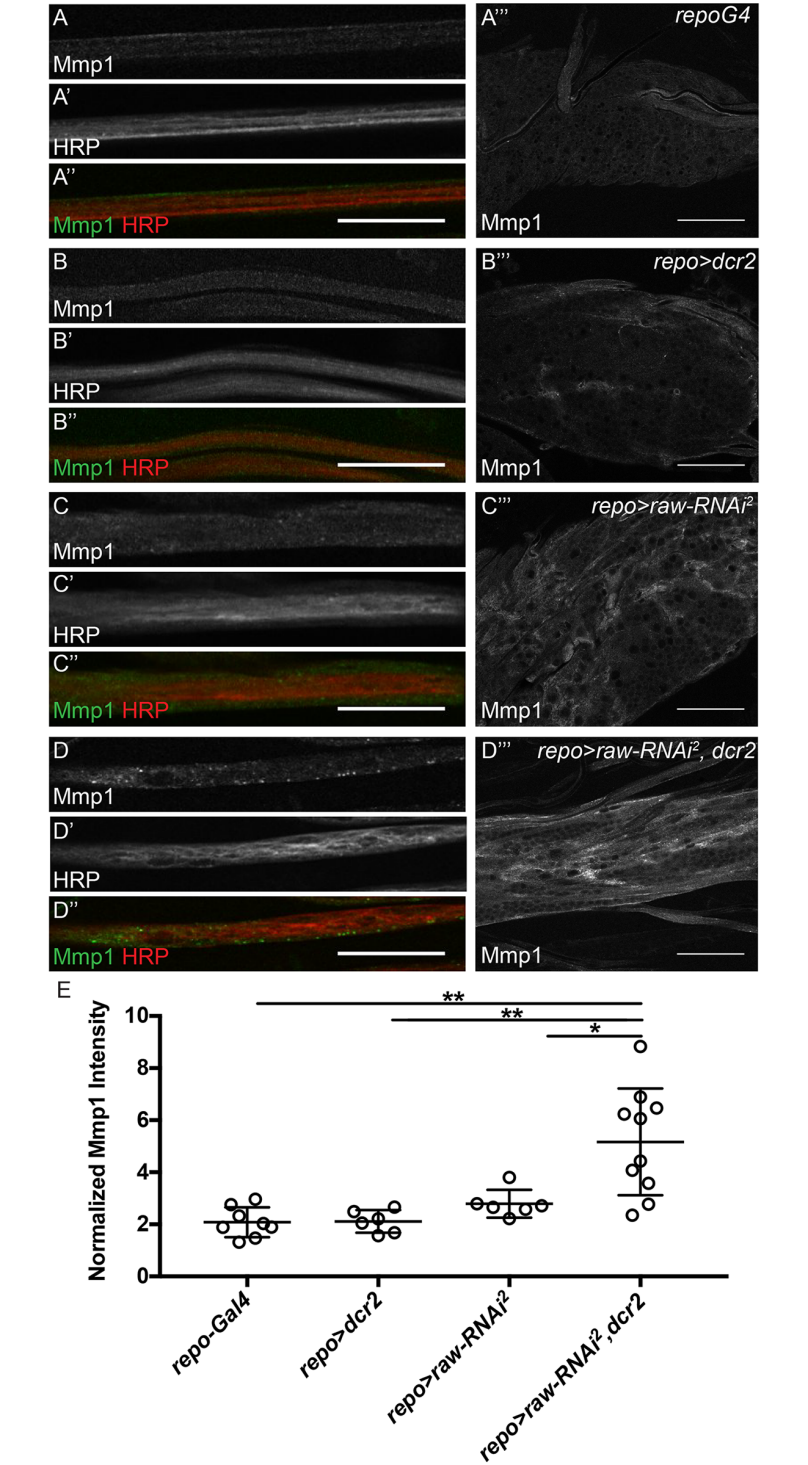
Mmp1 levels are increased upon *raw* knockdown. (A-D'') Single slices of peripheral nerves of third instar larvae co-stained for Matrix metalloproteinase 1 (anti-Mmp1, green) and neurons (anti-HRP, red). All images acquired with 60x oil objective with same confocal settings. Scale bars are 25μm. (A-D) Mmp1 alone. (A'-D') HRP alone. (A''-D'') Merge of Mmp1 (green) and neurons HRP (red) (A‴-D‴) Single slices of the ventral nerve cord (VNC) of third instar larvae stained for Mmp1. All images acquired with 60x oil objective with same confocal settings. Scale bars are 50μm. (A-A‴) Control animals (*repo-Gal4*; n = 9). (B-B‴) Control animals overexpressing *dcr2* (*repo-Gal4*> *dcr2*; n = 10). (C-C‴) Panglial *raw* knockdown (*repo-Gal4*>*raw-RNAi*^*2*^; n = 7). (D-D‴) Panglial *raw* knockdown in the presence of overexpressed *dcr2* results in an altered Mmp1 levels in nerves and the VNC (*repo-Gal4*>*raw-RNAi*^*2*^, *dcr2*; n = 10). Posterior to the right. (E) Quantitation of average Mmp1 intensity in VNC relative to adjacent muscle tissue. **p<0.001 and *p<0.01 based on one-way ANOVA with post-hoc Tukey's test.

Previous work has shown that increased levels of Matrix metalloproteinase 2 (Mmp2) in glia cause defects in VNC morphology, similar to the defects we observed upon *raw* knockdown [33]. Given that Raw has previously been demonstrated to negatively regulate JNK signaling in other contexts, and activation of JNK signaling results in increased transcription of *matrix metalloproteinase 1* (*mmp1*) [22, 24, 43], we hypothesized that *raw* knockdown may lead to increased Mmp1 levels. In the peripheral nerves, Mmp1-positive punctae were observed upon *raw* knockdown in the presence of overexpressed *dcr2*, but were absent from controls, demonstrating that Mmp1 localizes differently in nerves upon *raw* knockdown (Fig 6). In the VNC, immunostaining for Mmp1 upon *raw* knockdown revealed increased Mmp1 levels in the VNC relative to controls (Fig 6A‴ and 6C‴). These levels appeared further increased when *raw* was knocked down in the presence of overexpressed *dcr2* (Fig 6A‴–6D‴). Quantitation of normalized Mmp1 levels revealed significantly higher Mmp1 levels in the VNC of *raw* knockdown (*repo-Gal4*>*raw-RNAi*^*2*^, *dcr2*) samples compared to controls (*repo*-Gal4 and *repo-Gal4*>*dcr2*) (Fig 6E). From these results we conclude that *raw* knockdown leads increased JNK signaling and upregulation of the JNK target, *mmp1*.

**Fig 7:**
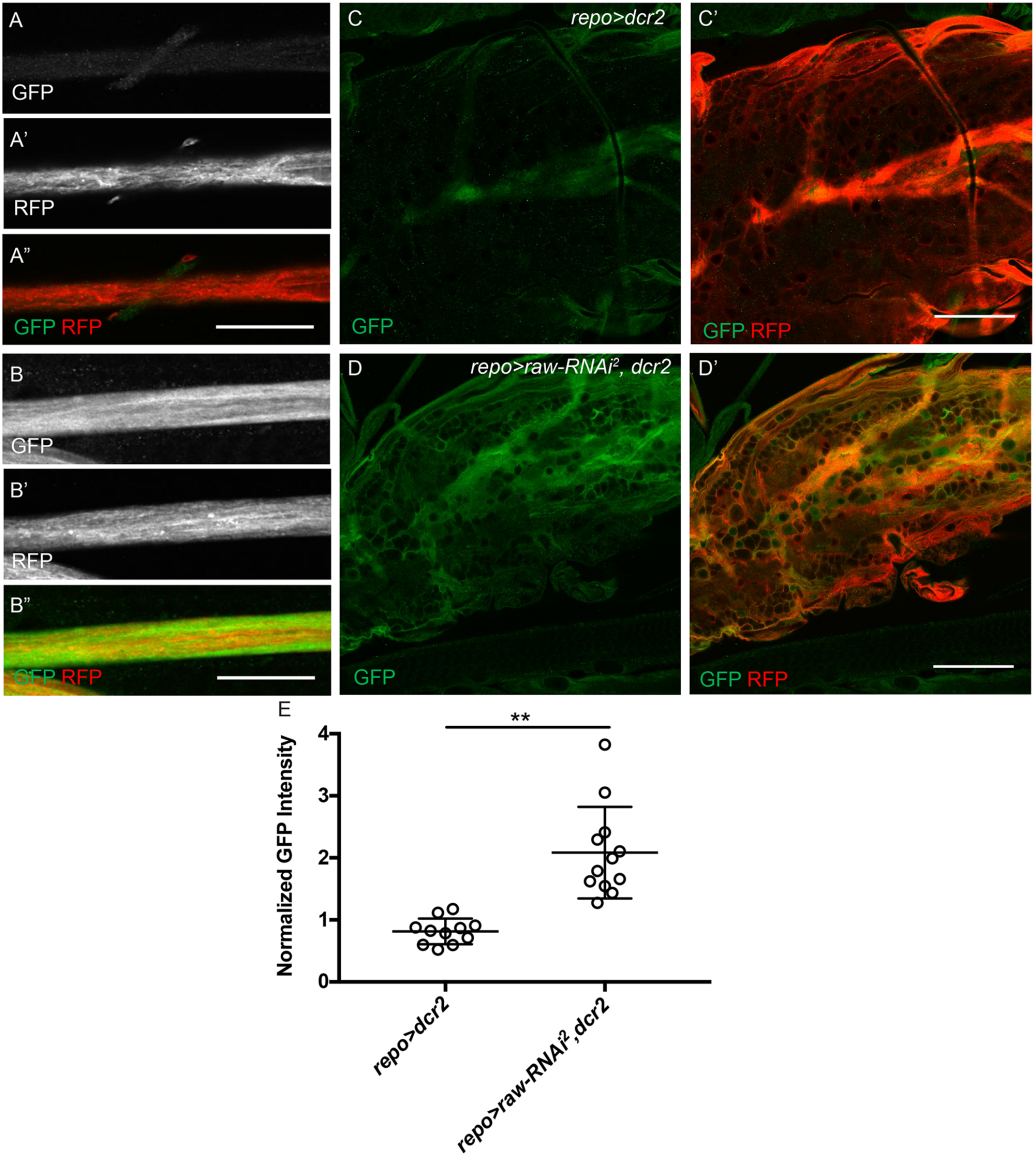
JNK reporter levels are increased upon *raw* knockdown. (A-B'') Single slices of peripheral nerves of third instar larvae co-stained for JNK Green Fluorescent Protein (GFP) reporter (green) and glial cytoplasm (Red Fluorescent Protein (RFP), red). All images acquired with 60x oil objective with same confocal settings. Scale bars are 25μm. (A-B) GFP alone. (A'-B') RFP alone. (A''-B'') Merge of GFP (green) and RFP (red) (C-D") Single slices of the ventral nerve cord of third instar larvae stained for GFP and RFP. All images acquired with 60x oil objective with same confocal settings. Scale bars are 50μm. (A-A", C-C') Control animals overexpressing *dcr2* (*repo-Gal4*> *dcr2*; n = 12). (B-B", D-D') Panglial *raw* knockdown in the presence of overexpressed *dcr2* results in an increase of GFP levels in nerves and the VNC (*repo-Gal4*>*raw-RNAi*^*2*^, *dcr2*; n = 13). Posterior to the right. (E) Quantitation of average GFP intensity in VNC relative to adjacent muscle tissue. **p < .0001 based on Mann-Whitney test.

While increased Mmp1 levels in *raw* knockdown samples suggests increased levels of JNK signaling, this increase could also occur indirectly through a different signaling pathway. Therefore, to demonstrate more directly that reducing *raw* levels increases JNK signaling, a JNK reporter (TRE-GFP) containing four binding sites for AP-1 (a heterodimeric Jun/Fos transcription factor targeted by JNK) upstream of GFP was utilized to examine JNK activity [27, 44, 45]. While GFP expression in the VNC and peripheral nerves was low in control samples (*repo-Gal4*>*dcr2*), a significant increase in GFP expression was observed when *raw* was knocked down in the presence of overexpressed *dcr2* (*repo-Gal4*>*raw-RNAi*^*2*^, *dcr2*) (Fig 7A–7D"). Quantitation of normalized expression levels revealed significantly higher GFP expression in the VNC in *raw* knockdown (*repo-Gal4*>*raw-RNAi*^*2*^, *dcr2*) samples as compared to the control (*repo-Gal4*>*dcr2*) (Fig 7E). From these results we conclude that *raw* knockdown leads to increased in JNK signaling and upregulation of downstream transcriptional targets.

### Increased JNK signaling results in reduced glial number

As JNK signaling is increased upon *raw* knockdown and glial cell number is reduced along peripheral nerves, it was hypothesized that simply increasing JNK signaling in glia could reduce glial number. Puckered (Puc) is a negative feedback regulator of JNK. Therefore, *puc* levels were reduced by RNAi to increase JNK signaling, and the number of glia along the A8/9 peripheral nerve was determined. Knockdown of *puc* resulted in an average of ~30 glia along the peripheral A8/9 nerve as compared to ~63 glia in the *repo*-Gal4 control (Fig 2H and 2I). These results suggest that increased JNK signaling negatively impacts glial cell number, and that that effect of *raw* knockdown on glial number could occur as a result of a failure of Raw to inhibit JNK signaling in glia.

### *raw* knockdown in glia affects locomotor function

**Fig 8:**
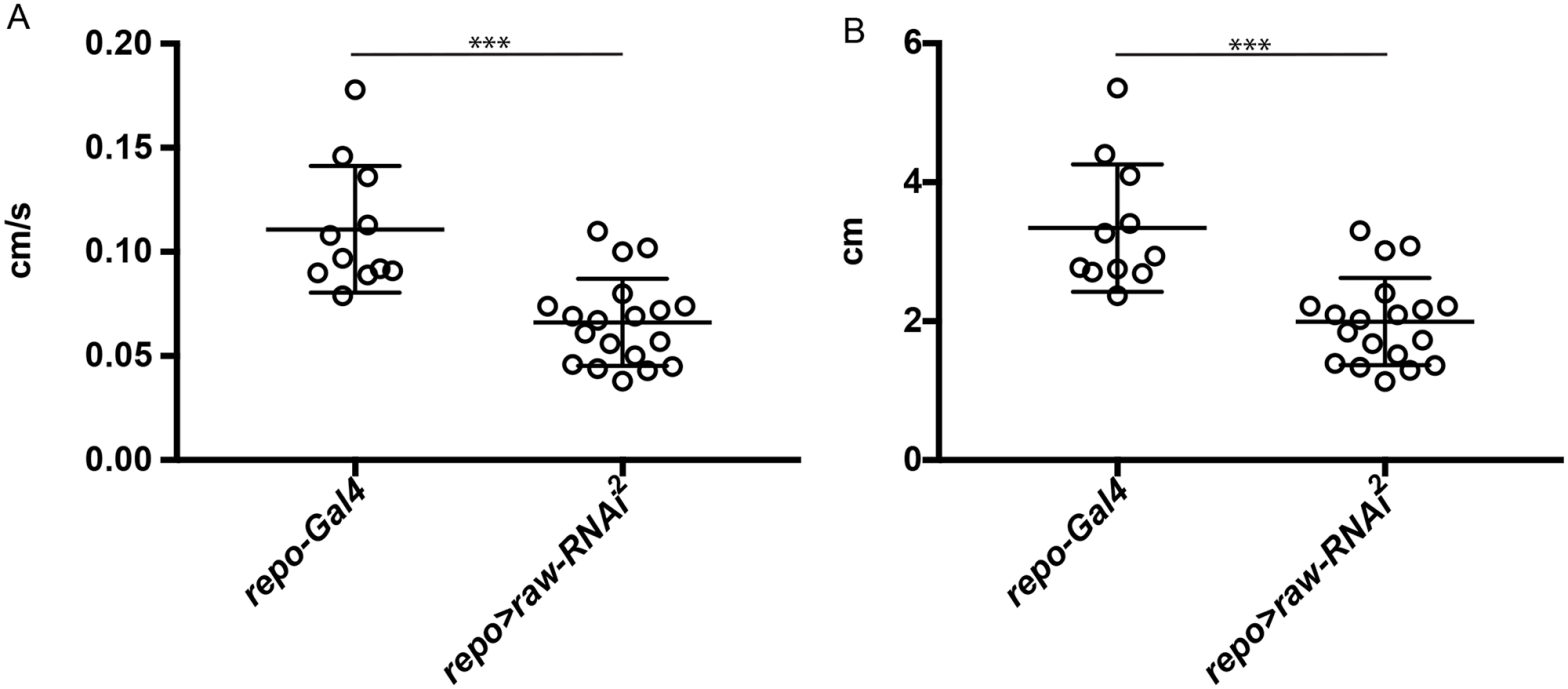
*raw* knockdown in glia results in reduced larval crawling. (A) Crawling speed assayed in control animals (*repo-Gal4*; n = 11) and *raw* knockdown animals (*repo-Gal4*, UAS-*raw-RNAi*^*2*^; n = 19) over a 30 second interval. p<0.0001 based on unpaired, two-tailed t-test. (B) Distance traveled by control animals (*repo-Gal4*; n = 11) and *raw* knockdown animals (*repo-Gal4*>*raw-RNAi*^*2*^; n = 19) over a 30 second interval. ***p<0.0001 based on Mann-Whitney test.

The defects observed in glial number and nervous system morphology raised the possibility that overall nervous system function may be disrupted. In order to examine nervous system function, larval crawling assays were performed. The average speed and distance traveled by larvae over a period of 30 seconds were analyzed. Control larvae (*repo*-Gal4) crawled at an average speed of .11cm/s, whereas *repo>raw-RNAi larvae* crawled at an average speed of 0.066cm/s (Fig 8A; S1 and S2 Movies). The average distance covered by controls was also significantly greater at 3.34cm, as compared to 1.99cm for *repo>raw-RNAi larvae* (Fig 8B). These results demonstrate that reduced *raw* levels not only affect glial cell number, but also result in impaired locomotor activity.

## Discussion

Our studies have resulted in the identification of Raw as a regulator of glial development. We show that reducing *raw* levels in glia affects the morphology of the CNS, resulting in a smaller brain and an elongated VNC. In addition, examination of glial cell number along the peripheral nerves revealed that *raw* knockdown led to a reduction in the number of glia along the A8/9 peripheral nerves, which is due in part to reduced glial proliferation. Previous studies demonstrated that Raw functions as a negative regulator of JNK signaling in other developmental contexts. Therefore, we examined the expression of a JNK reporter and Mmp1, a downstream target of JNK signaling. We observed increased reporter expression and Mmp1 levels in the VNC and increased reporter expression and Mmp1-positive punctae along peripheral nerves upon *raw* knockdown in the presence of overexpressed *dcr2*. Supporting the hypothesis that increased JNK signaling can lead to defects in glial development, knockdown of *puc*, a negative feedback regulator of the JNK pathway, also resulted in a decrease in the number of glia along peripheral nerves. Finally, larvae with reduced *raw* levels had reduced crawling ability, suggesting that reduced glial number negatively impacts motor neuron function. These results suggest that Raw functions as a negative regulator of JNK signaling in the context of glial development, thereby affecting glial number and leading to morphological defects that impair nervous system function.

### Molecular role of Raw and JNK signaling in gliogenesis

Embryonic studies have provided insight into how Raw regulates JNK signaling, although our understanding remains far from complete. Recent work revealed that Raw regulates expression of the long non-coding RNA *acal*, which functions to negatively regulate expression of *Connector of kinase to AP-1* (*Cka*), a protein that acts as a scaffold for JNK and *Drosophila* Jun, known as Jun-related antigen (Jra) [46, 47]. These results are consistent with earlier studies, suggesting that Raw functions at the level of the JNK, Basket (Bsk), and limits accumulation of Jra in the nucleus [20, 22, 24]. Thus, Raw may function to reduce efficiency of JNK signaling by limiting scaffold production in glia as well.

While previous studies have demonstrated that JNK signaling is upregulated in glia in response to neuronal injury and plays a role in promoting glial phagocytosis of neurons during development, the role of JNK signaling in gliogenesis has been largely unexplored [48–50]. Studies in the embryo showed that mutation of *zinc finger homeodomain 1* (*zfh1*) promoted apoptosis of ePG10 in a JNK-dependent manner, although Zfh1 was expressed in all ePGs [51]. Our results demonstrate that *raw* knockdown increases expression of a JNK reporter and the JNK target, *mmp1*, and that increased JNK signaling in glial cells results in a significant decrease in the number of glia along peripheral nerves, similar to that observed upon *raw* knockdown. These results suggest that it is altered JNK signaling that is responsible for the reduction in glial number observed upon *raw* knockdown rather than regulation of another signaling pathway.

However, Raw may also function with other molecular players to regulate glial development, as JNK signaling is not the only pathway with which Raw has been shown to interact. In class IV dendritic arborization neurons, Raw appears to function independently of JNK signaling and instead cooperates with Trc, a NDR family kinase, to regulate dendritic patterning [19]. NDR family kinases include Trc and Warts (Wts), which each belong to different NDR kinase subfamilies, and have been demonstrated to function downstream of the protein kinase Hippo (Hpo) [52, 53]. Wts negatively regulates activity of the transcription factor Yorkie (Yki), and thereby limits cell proliferation [52, 54]. Interestingly, activation of Yki is required for glial proliferation in the context of the brain and developing eye in *Drosophila* [55]. In contrast to Wts, Trc appears to function through downstream effectors other than Yki [52, 54]. In fact, the mammalian homolog of Trc, Ndr1, promotes progression through the cell cycle via phosphorylation of the cell cycle inhibitory protein p21 [56]. These results suggest that Wts and Trc have distinct functions downstream of Hpo. Examination of the roles of Wts and Trc in dendrites supports this conclusion, as Trc regulates dendritic tiling, while Wts functions in dendritic maintenance [53]. Studies in the context of dendrite morphogenesis suggest Trc functions via Rac GTPase to regulate the actin cytoskeleton, suggesting a mechanism by which Raw and Trc could function to promote changes in nervous system morphology during development [57]. Given the proliferation defects observed upon *raw* knockdown, and the previously characterized interaction of Raw and Trc, it raises the possibility that Raw may be functioning in cooperation with NDR family kinases to regulate glial proliferation and nervous system morphology.

While the above results suggest Raw may function in the context of Hpo signaling, it is important to note that the Hpo and JNK signaling pathways have also been observed to regulate each other's function. In *Drosophila* models of tumorigenesis, activation of Hpo has been observed to increase JNK signaling to promote cell invasion [58], while JNK signaling has been demonstrated to enhance Yki activation by inhibition of Wts [59, 60]. However, in normal epithelium, JNK signaling has been observed to inhibit Yki activation [60]. These results suggest the mechanisms by which these pathways influence each other is context-dependent. Given that Yki is a positive regulator of glial proliferation, the relationship between JNK signaling and Yki activation in normal epithelium would be consistent with a model in glia where *raw* knockdown results in increased JNK signaling, leading to a decrease in Yki activation and reduced glial proliferation. [55].

### Regulation of glial development and function

In addition to understanding the molecular context of Raw function, it is also important to identify the specific glial subtypes that require Raw activity, and to understand the role of this activity in cell-cell and cell-matrix interactions. Previous work demonstrated that expression of Mmp2 in all glia (excluding the midline glia) or the perineurial glia can lead to an elongated VNC, while panglial overexpression of Mmp1 was larval lethal [12, 33]. Our findings that *raw* knockdown results in increased levels of Mmp1 and an elongated VNC are consistent with a potential requirement for Raw function in mediating the interaction of perineurial glia with the surrounding neural lamella. Raw has been demonstrated to regulate dendrite-ECM adhesion in cooperation with Trc, again suggesting a similar regulatory mechanism could exist in glia [19]. In mammals, loss of cell-ECM interactions results in increased Hpo signaling and inhibition of YAP, the mammalian homolog of Yki [61]. Thus, the increase in Mmp1 levels observed upon *raw* knockdown could reduce glia-matrix interactions, leading to reduced perineurial glial proliferation. Future exploration of the relationship of Raw to components of the Hpo signaling pathways is critical for our understanding of how Raw regulates glial development.

While these data suggest a cell autonomous role for Raw in perineurial glia, is does not exclude the possibility that Raw could function cell non-autonomously to regulate perineurial glia proliferation, or that Raw may have additional cell autonomous functions in the subperineurial and wrapping glia. The fact that larvae have motor defects also supports the possibility that Raw functions in the glia that are more tightly associated with motor neurons to ensure their proper function. Thus, knockdown of *raw* in each glial subtype is critical for addressing these possibilities. Examination of the ability of Raw to interact with other signaling pathways during development will increase our understanding of the mechanisms regulating gliogenesis, and may also provide insight into the underlying causes of neuropathy and neurodegenerative disease.

## Supporting information

S1 Fig Phospho-Histone H3 staining in the eye disc. Third instar eye-antennal imaginal disc immunostained with pH3 (green) to identify cells in mitosis and Elav (red) for photoreceptor neurons. pH3 immunostaining recapitulates previously published data. Scale bar is 100μm.
(TIF)

S1 Movie Larval crawling of control larvae (*repo-Gal4*; n = 11) over a 30 second interval. (AVI)

S2 Movie Larval crawling of *raw* knockdown animals (*repo-Gal4*, UAS-*raw-RNAi*^*2*^; n = 19) over a 30 second interval. (AVI)
