# Supplementary figures and images for: Identification of raw as a regulator of glial development
Source: PLoS One. 2018 May 29;13(5):e0198161. doi: 10.1371/journal.pone.0198161 (PMC5973607; doi:10.1371/journal.pone.0198161)

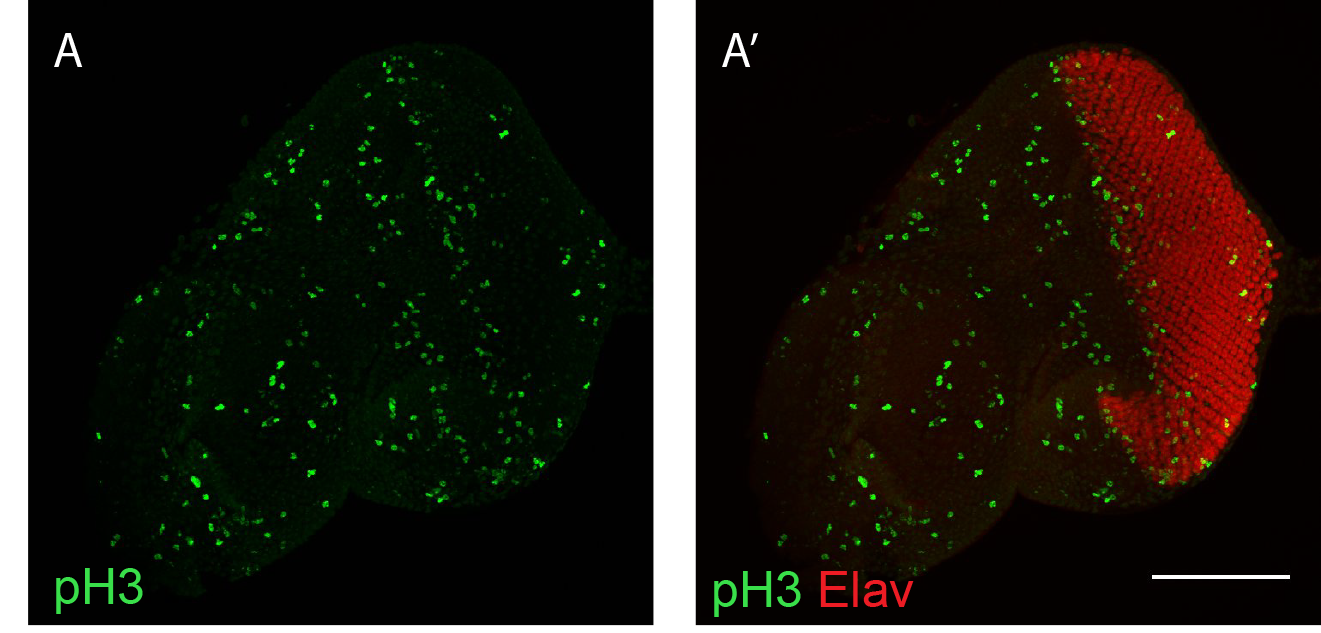

Supplement: S1 Fig — Third instar eye-antennal imaginal disc immunostained with pH3 (green) to identify cells in mitosis and Elav (red) for photoreceptor neurons. pH3 immunostaining recapitulates previously published data. Scale bar is 100μm. (TIF) [file pone.0198161.s001.tif]
